# Liensinine and Nuciferine, Bioactive Components of* Nelumbo nucifera*, Inhibit the Growth of Breast Cancer Cells and Breast Cancer-Associated Bone Loss

**DOI:** 10.1155/2017/1583185

**Published:** 2017-12-03

**Authors:** Eun Ji Kang, Sun Kyoung Lee, Kwang-Kyun Park, Seung Hwa Son, Ki Rim Kim, Won-Yoon Chung

**Affiliations:** ^1^Department of Oral Biology, Oral Cancer Research Institute, BK21 PLUS Project, Yonsei University College of Dentistry, Seoul 03722, Republic of Korea; ^2^Department of Applied Life Science, The Graduate School, Yonsei University, Seoul 03722, Republic of Korea; ^3^Department of Dental Hygiene, Gangdong College, Icheon 27600, Republic of Korea; ^4^Department of Dental Hygiene, Kyungpook National University, Sangju 37224, Republic of Korea

## Abstract

Once breast cancer cells grow aggressively and become lodged in the skeleton through migration and invasion, they interact with bone microenvironment and accelerate much more tumor growth and bone destruction. We investigated whether liensinine and nuciferine, major active components in* Nelumbo nucifera* (lotus), could prevent breast cancer cell-mediated bone destruction. Liensinine and nuciferine inhibited the growth of MDA-MB-231 and MCF-7 human breast cancer cells by inducing apoptosis and inhibiting proliferation via cell cycle arrest. Liensinine treatment led to the increased Bax/Bcl-2 ratio, activation of caspase-3, and subsequent cleavage of PARP. Liensinine also displayed significant inhibition on the migration and invasion of both MDA-MB-231 and MCF-7 human breast cancer cells compared with nuciferine. In addition, liensinine and nuciferine inhibited the receptor activator of nuclear factor kappa-B ligand- (RANKL-) induced osteoclast differentiation in mouse bone marrow macrophage cells and mature osteoclast-mediated bone resorption. Furthermore, oral administration of liensinine reduced the osteolysis in nude mice with intratibial injection of MDA-MB-231 cells. Collectively, liensinine and nuciferine may be promising candidates for preventing and treating breast cancer bone metastasis and the resulting osteolytic bone loss by targeting both cancer cells and osteoclasts. Liensinine has more potent anticancer and antibone resorptive activities than nuciferine.

## 1. Introduction

Breast cancer is the common form of cancer in women and the major cause of cancer deaths worldwide, accounting for 23% of cancer diagnoses and 14% of cancer deaths each year [1]. Although surgical therapies removing primary breast cancer have shown beneficial effect, they are not fundamental solution because most causes of morbidity in breast cancer are not primary tumors but incurable complications from bone metastasis, including pathologic fractures, disability, pain, nerve compression, anemia, and hypercalcemia [2, 3].

Bone is a dynamic organ that continuously undergoes remodeling processes to maintain mineral homeostasis and structural robustness [4]. Normal bone remodeling is finely regulated by balance between osteoblastic bone formation and osteoclastic bone resorption [5]. Once metastatic breast cancer cells enter into the bone microenvironment, they disturb the normal regulatory mechanisms associated with bone remodeling process by inducing activation of bone-resorbing osteoclasts [6]. Breast cancer-induced factors stimulate osteoblastic/stromal cells to produce macrophage-colony-stimulating factor (M-CSF) for the survival of osteoclast precursors and receptor activator of nuclear factor-*κ*B ligand (RANKL) for osteoclastogenesis [7]. Mature bone-resorbing osteoclasts secrete hydrogen ion for acidic bone microenvironment and proteases for the degradation of the organic bone matrix. Currently, resorption inhibitors, such as bisphosphonates and denosumab, are clinically used to treat the patients with bone metastases. However, these agents targeting osteoclastic activity do not prevent the development of bone metastasis and ultimately do not prolong survival [8].

**Figure 1:**
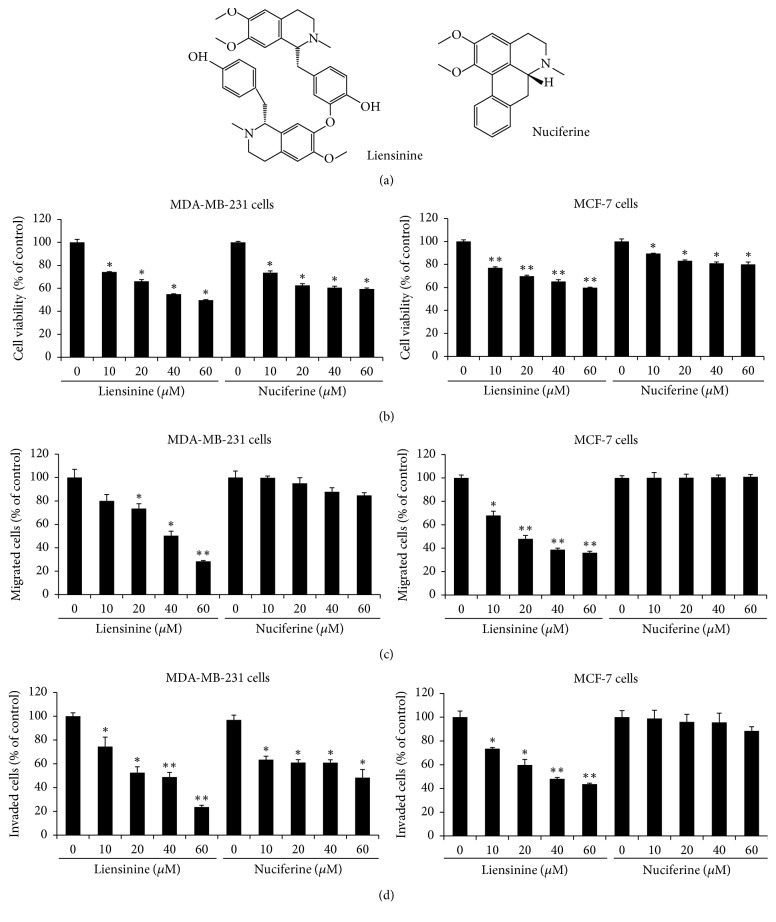
Liensinine and nuciferine inhibited the viability, migration, and invasion of breast cancer cells. (a) Chemical structures of liensinine and nuciferine. (b) MDA-MB-231 or MCF-7 cells were treated with various concentrations of liensinine and nuciferine in serum-free media for 24 h. Cell viability was determined by an MTT assay. (c) In the presence of liensinine or nuciferine at the indicated concentrations, MDA-MB-231 or MCF-7 cells were added to transwell chamber and attracted by 5% FBS for 24 h. (d) MDA-MB-231 or MCF-7 cells were seeded into the matrigel-based upper chamber with serum-free media containing liensinine or nuciferine. The lower chamber was filled with 600 *μ*l of media containing 5% FBS and liensinine or nuciferine for 24 h. Data are expressed as the mean ± SE. ^*∗*^*P* < 0.05 and ^*∗∗*^*P* < 0.001 versus untreated cells.

Liensinine and nuciferine (Figure 1(a)) are principal components in lotus (*Nelumbo nucifera* Gaertn, Nymphaeaceae), which is extensively cultivated in Eastern Asia, particularly in China, and has been used as remedy for the disorders associated with oxidative stress, metabolic syndrome, immunity, and inflammation [9–11]. Liensinine, a bisbenzylisoquinoline alkaloid, has been reported to inhibit autophagy and to increase apoptosis in breast cancer cells cotreated with various chemotherapeutic agents [12]. Isoliensinine treatment caused apoptosis through the production of reactive oxygen species and p38 MAPK/JNK activation in triple-negative human breast cancer cells [13]. Nuciferine, an aporphine alkaloid, has been shown to reduce the viability of SY5Y human neuroblastoma cells and CT26 murine colon cancer cells and to inhibit tumor growth in nude mice xenografted with these cancer cell lines [14]. In addition, nuciferine inhibited nicotine-induced non-small-cell lung cancer progression [15].

In the current study, we attempted to estimate whether liensinine and nuciferine could prevent breast cancer-mediated bone destruction by examining their effects on the growth, motility, and invasiveness of human triple-negative MDA-MB-231 and human estrogen receptor-positive MCF-7 cells, RANKL-induced osteoclast differentiation in bone marrow macrophages (BMMs), and mature osteoclast-mediated bone resorption. Furthermore, the inhibitory effect of liensinine on the production of breast cancer-induced osteolytic lesions was determined in mice with intratibial injection of MDA-MB-231 breast cancer cells.

## 2. Materials and Methods

### 2.1. Materials

Liensinine and nuciferine* (≥98% by HPLC)* were purchased from* ChemFaces (Wuhan, Hubei, China)* and dissolved with dimethyl sulfoxide (DMSO) and ethanol, respectively. Dulbecco's modified Eagle medium (DMEM), minimum essential medium alpha (*α*-MEM), Dulbecco's phosphate-buffered saline (PBS), fetal bovine serum (FBS), and antibiotic-antimycotic mixture containing 100 U/ml penicillin and 100 *μ*l/ml streptomycin were obtained from Gibco BRL (Grand Island, NY). 3-(4,5-Dimethylthiazol-2-yl)-2,5-diphenyltetrazolium bromide (MTT), histopaque-1083, and DMSO were purchased from Sigma-Aldrich (St. Louis, MO). Anti-human Bcl-2, procaspase-3, caspase-3, poly(ADP-ribose) polymerase (PARP), and RANKL antibodies were obtained from Cell Signaling Technology (Beverly, MA). Anti-human Bax and horseradish peroxidase- (HRP-) conjugated anti-rabbit second antibodies were obtained from Santa Cruz Biotechnology (Santa Cruz, CA). Recombinant mouse soluble RANKL (sRANKL) and M-CSF were purchased from R&D system (Minneapolis, MN). All reagents used in this study were of analytical grade.

### 2.2. Animals

5-week-old female Balb/c* nu*/*nu* mice and 4-week-old male ICR mice were obtained from the Nara Biotechnology (Seoul, Korea). All mice were provided with free access to a commercial rodent chow diet and tap water and maintained under specific pathogen-free conditions with a 12 h light-dark cycle at 22 ± 2°C. All animal experimental procedures were conducted in compliance with the guidelines and regulations for the use and care of animals established by Yonsei University College of Dentistry. All methods were carried out in accordance with relevant guidelines and regulations.

### 2.3. Cell Lines and Cell Cultures

Human breast cancer cells lines, MDA-MB-231 and MCF-7, were obtained from the Korean Cell Line Bank (Seoul, Korea) and grown in DMEM medium supplemented with 10% FBS at 37°C under a humidified atmosphere of 5% CO_2_. Mouse BMMs isolated from the tibiae of 4-week-old ICR mice were cultured with *α*-MEM containing M-CSF (30 ng/ml), 10% FBS, and 1% antibiotic-antimycotic mixture in a humidified atmosphere of 5% CO_2_ at 37°C.

### 2.4. Cell Viability Assay

MDA-MB-231 or MCF-7 cells (5 × 10^3^ cells/well) were cultured in serum-free media with the various concentrations of liensinine or nuciferine in the absence or presence of zVAD-fmk (10 *μ*M) for 24 h. BMMs (5 × 10^4^ cells/well) were cultured in serum-free medium with liensinine or nuciferine at the indicated concentrations for 5 days. Cell viability was measured with an MTT assay.

### 2.5. Transwell Migration and Invasion Assay

Cell migration and invasion were detected using transwell chamber (Corning Life Sciences, Corning Costar, MA). For cell migration assay, the chamber was coated with 10% (w/v) gelatin on lower surface of polycarbonate membrane (6.5 mm in diameter, 8 *μ*m pore size). For cell invasion assay, the chamber was coated with 10% (w/v) gelatin on lower surface and matrigel (1 mg/ml in distilled water) on upper surface of polycarbonate membrane. The upper chamber was filled with MDA-MB-231 (5 × 10^4^ cells/100 *μ*l) or MCF-7 (1 × 10^5^ cells/100 *μ*l) cell suspension and serum-free media containing different concentrations of liensinine or nuciferine. The lower chamber contained 600 *μ*l of DMEM containing 5% FBS and various concentrations of liensinine or nuciferine. After 24 h of incubation, migrated or invaded cells at the lower surface of the membrane were counted as previously described [16, 17].

### 2.6. Apoptosis and Necrosis Assay

Apoptotic and necrotic cell death was measured using the Cell Death Detection ELISA Kit (Roche Diagnostics, Mannheim, Germany) according to the manufacturer's instruction. Briefly, MDA-MB-231 or MCF-7 cells (5 × 10^3^ cells/well) were treated with the various concentrations of liensinine or nuciferine in serum-free media for 24 h. After centrifugation at 200 ×g for 10 min, the supernatants were collected and the cells were incubated with lysis buffer for 30 min at room temperature. The supernatants and cell lysates were incubated in the anti-histone antibody-coated wells of a microplate for 2 h. Absorbance was measured at 405 nm using a Benchmark Microplate Reader.

### 2.7. Cell Proliferation Assay

MDA-MB-231 or MCF-7 cells (5 × 10^3^ cells/well) were treated with the various concentrations of liensinine or nuciferine in serum-free media for 24 h. The amount of incorporated 5-bromo-2-deoxyuridine (BrdU) into newly synthesized DNA of proliferating cells was detected using a BrdU cell proliferation assay kit (Roche Applied Science, Penzberg, Germany). Absorbance was measured at a wavelength of 450 nm in a Benchmark Microplate Reader.

### 2.8. Cell Cycle Distribution Analysis

MDA-MB-231 or MCF-7 cells (5 × 10^5^ cells) were treated with different concentrations of liensinine or nuciferine in serum-free media for 24 h. The cells were harvested, washed with PBS, and fixed with ice-cold 70% ethanol overnight at 4°C. The cells were washed with PBS again and incubated in solution containing 1 mg/ml RNase A, 0.1% Triton-X, and 0.1 mg/ml propidium iodide at room temperature in the dark for 30 min. DNA content was then analyzed using flow cytometer (BD Bioscience, Franklin Lakes, NJ).

### 2.9. Western Blot Analysis

MDA-MB-231 or MCF-7 cells in 100 mm culture dishes were treated with different concentrations of liensinine in serum-free media for 24 h and then lysed with RIPA buffer (Cell Signaling Technology) containing 1 mM phenylmethylsulfonyl fluoride and protease inhibitor cocktail (Roche, Mannheim, Germany). Cell lysates were centrifuged at 22,000 ×g for 15 min at 4°C and the protein concentration in the supernatant was determined using the BCA protein assay kit (Pierce, Rockford, IL). Equal amounts of protein (40 *μ*g) were subjected to electrophoresis on sodium dodecyl sulfate- (SDS-) polyacrylamide gels and the blots on the gel were transferred to the polyvinylidene difluoride membrane (Millipore, Billerica, MA). The membranes were blocked with 10% skim milk for 1 h at room temperature and then were incubated with the specific primary antibody against target proteins in 3% skim milk at a dilutions specified by the manufacturer. After washing with TBS-T (10 mM Tris, pH 8.0, 150 mM NaCl, and 0.1% Tween-20), the membrane was incubated with the respective horseradish peroxidase-conjugated secondary antibody at 1 : 2,000 dilutions for 1 h at room temperature. The target protein was detected using Enhanced Chemiluminescence kit (Amersham, Piscataway, NJ) according to manufacturer's protocol.

### 2.10. Osteoclast Formation Assay

BMMs (5 × 10^4^ cells/well) were incubated with *α*-MEM containing 10% FBS, M-CSF (30 ng/ml), sRANKL (100 ng/ml), and different concentrations of liensinine or nuciferine for 5 days. The media were replaced with fresh media every second day. The cells were stained using the Acid Phosphatase Leukocyte kit (Sigma-Aldrich) and tartrate-resistant acid phosphatase- (TRAP-) positive multinucleated osteoclasts with more than three nuclei were counted under light microscopy (×100 magnification) as described previously [16, 17].

### 2.11. Pit Formation Assay

BMMs (5 × 10^4^ cells/well) were seeded into an Osteo assay plate (Corning, NY) and cultured with *α*-MEM containing 10% FBS, M-CSF (30 ng/ml), and sRANKL (100 ng/ml) for 5 days. BMMs were then treated with liensinine or nuciferine at the indicated concentrations for 2 additional days. The activities of cathepsin K and matrix metalloproteinases (MMPs) in the collected media were measured using SensiZyme cathepsin K assay kit (Sigma-Aldrich) and gelatin zymography, respectively, and the resorbed pits on the plate were observed under light microscope (×100 magnification), as previously described [16, 17].

### 2.12. A Murine Model of Breast Cancer-Induced Osteolysis

Female Balb/c* nu/nu* mice were randomly divided into 6 groups, with 6 mice per group. The mice were anesthetized by intraperitoneal injection of a mixture with 30 mg/kg Zoletil (Virbac Laboratories, Carros, France) and 10 mg/kg Rompun (Bayer HealthCare Korea, Seoul, Korea) mixture. MDA-MB-231 cells (1 × 10^6^ cells/0.5 ml HBSS) were injected into the medullar cavity of the proximal tibia of the mice using a Hamilton syringe and 27-gauge needle. From the next day, liensinine at 5, 10, or 20 mg/kg of body weight (BW) was orally administered 5 times per week and zoledronic acid at 0.1 mg/kg BW was subcutaneously injected 3 times per week for 5 weeks. Control and MDA-MB-231 cell alone-inoculated mice received* vehicle (PBS containing 1% DMSO)* instead of liensinine. For histological examination, tibiae and femora of mice were fixed in 10% buffered formalin solution at 4°C for a week. Tibiae and femora were decalcified with 10% EDTA solution (pH 7.5) at 4°C for two weeks and then embedded in paraffin. Serial sections (3–5 *μ*m thick) were stained with hematoxylin-eosin (H&E) as describe previously [18]. The tibia of mice was analyzed with microcomputed tomography (micro-CT) system (SkyScan 1076, SkyScan, Kontich, Belgium) with 100 kV, 140 *μ*A current, and rotation step 0.6°. Scans were reconstructed in the NRecon software (SkyScan). Bone morphometric parameters were analyzed in the proximal tibiae of nude mice using CTAn software (SkyScan). The serum levels of tartrate-resistant acid phosphatase (TRAP) 5b and C-terminal cross-linking telopeptide of type I collagen (CTX) were quantified using mouse TRAP assay kit (Immunodiagnostic Systems, Boldon, UK) and RatLaps EIA kit (Immunodiagnostic Systems) according to the manufacturer's instructions, respectively.

### 2.13. Statistics Analysis

Data were expressed as mean ± standard error (SE) of three independent experiments. Statistical analysis was performed with a one-way ANOVA and Student's *t*-test to express the difference between the two groups. If the *P* value is under 0.05, results are considered statistically significant.

## 3. Results

### 3.1. Liensinine and Nuciferine Decreased the Viability, Migration, and Invasion of Breast Cancer Cells

When MDA-MB-231 and MCF-7 cells were incubated with increasing doses of liensinine and nuciferine for 24 h, their viability was decreased in a dose-dependent manner (Figure 1(b)). Liensinine and nuciferine at 60 *μ*M reduced cell viability by 50% and 40% in MDA-MB-231 cells and by 40% and 20% in MCF-7 cells, respectively. In addition, the migration assay using transwell chamber indicated that liensinine at 60 *μ*M inhibited cell migration by 72% in MDA-MB-231 cells and by 61% in MCF-7 cells (Figure 1(c)). However, nuciferine did not show significant inhibition in both cell lines. The invasion of MDA-MB-231 and MCF-7 cells was inhibited by 76% and 56% by liensinine treatment at 60 *μ*M, respectively. Nuciferine treatment at 60 *μ*M reduced the invasion of MDA-MB-231 cells by 52% but not that of MCF-7 cells (Figure 1(d)).

### 3.2. Liensinine and Nuciferine Induced the Apoptosis and Inhibited Cell Proliferation in Breast Cancer Cells

**Figure 2:**
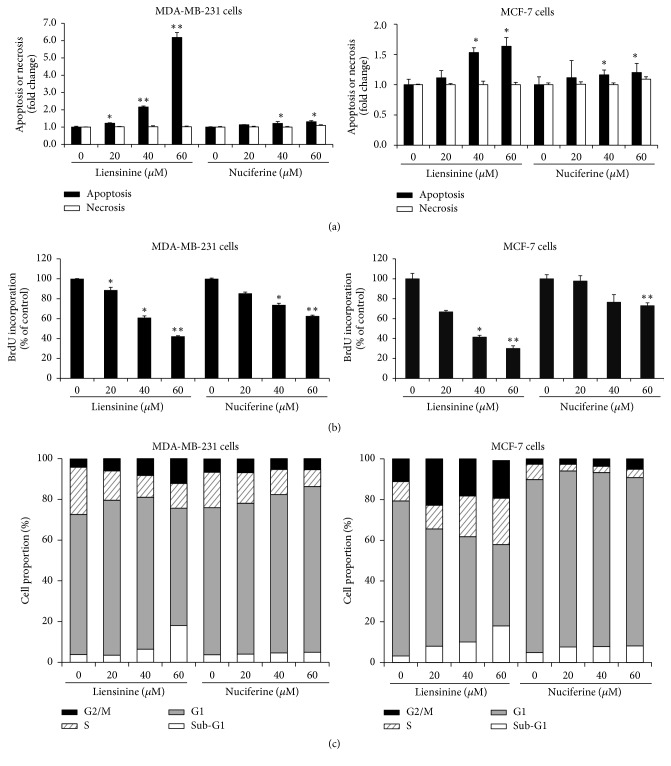
Liensinine and nuciferine induced apoptosis and inhibited the proliferation in breast cancer cells. MDA-MB-231 or MCF-7 cells were treated with various concentrations of liensinine and nuciferine in serum-free media for 24 h. (a) Apoptosis and necrosis were evaluated with Cell Death Detection ELISA to detect DNA fragments in culture supernatants and cells lysates. (b) The amount of newly synthesized DNA was assessed by BrdU incorporation assay. (c) The cells were fixed and stained with propidium iodide. The cell cycle distribution was analyzed by flow cytometry. Data are expressed as the mean ± SE. ^*∗*^*P* < 0.05 and ^*∗∗*^*P* < 0.001 versus untreated cells.

To investigate whether the liensinine and nuciferine could induce apoptosis in MDA-MB-231 and MCF-7 cells, histone-associated DNA fragments were measured in lysate (apoptosis) and culture supernatants (necrosis) of the cells treated with the various concentrations of liensinine and nuciferine for 24 h using the Cell Death Detection ELISA Kit. Liensinine treatment induced apoptosis in a dose-dependent manner and sharply increased particularly at 60 *μ*M in MDA-MB-231 cells. In contrast, apoptosis was slightly induced in liensinine-treated MCF-7 cells and nuciferine-treated breast cancer cells (Figure 2(a)). Treatment with liensinine and nuciferine at 60 *μ*M and less did not cause necrosis in two breast cancer cell lines.

Next, we examined the inhibitory effects of liensinine and nuciferine on cell proliferation by measuring the amount of incorporated BrdU into DNA in MDA-MB-231 and MCF-7 cells treated with each compound for 24 h. Liensinine and nuciferine inhibited the incorporation of BrdU in these breast cancer cells in a dose-dependent manner. Liensinine at 60 *μ*M decreased BrdU incorporation by 58% and 70% in MDA-MB-231 and MCF-7 cells, respectively. Nuciferine at 60 *μ*M reduced BrdU incorporation by 37% and 27% in MDA-MB-231 and MCF-7 cells, respectively (Figure 2(b)).

We further analyzed cell cycle distribution in breast cancer cells treated with liensinine and nuciferine in serum-free media for 24 h. Flow cytometric analysis indicated that treatment with liensinine dose-dependently increased sub-G1 peak as an indicator of apoptotic cell death and cell population at G2/M phase in MDA-MB-231 and MCF-7 cells. Nuciferine slightly increased sub-G1 peak in both cell lines but induced G1 arrest in MDA-MB-231 cells and G2 arrest in MCF-7 cells. However, nuciferine did not show substantial effect (Figure 2(c)).

### 3.3. Liensinine Induced Caspase-Dependent Apoptosis in Breast Cancer Cells

**Figure 3:**
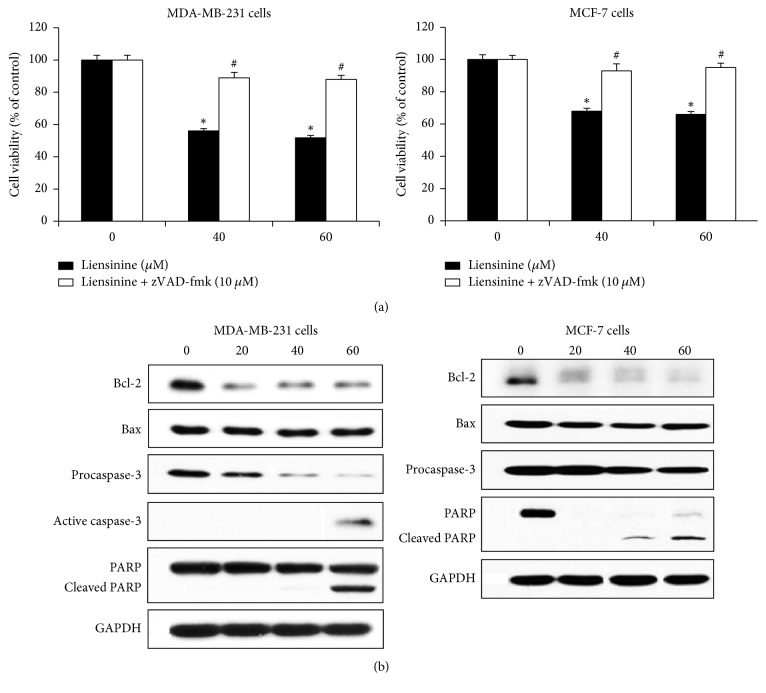
Liensinine inhibited Bcl-2 expression and induced apoptosis via caspase-dependent pathway in breast cancer cells. (a) MDA-MB-231 or MCF-7 cells were cultured in serum-free media with the various concentrations of liensinine in the absence or presence of zVAD-fmk (10 *μ*M) for 24 h. Cell viability was determined with an MTT assay. Data are expressed as the mean ± SE. ^*∗*^*P* < 0.01 versus untreated cells and ^#^*P* < 0.01 versus liensinine alone-treated cells. (b) MDA-MB-231 and MCF-7 cells were treated with various concentrations of liensinine for 24 h. Cell lysates were prepared and the levels of Bcl-2, Bax, procaspase-3 and active caspase-3, and full length and cleaved PARP were detected by western blotting.

We attempted to verify the molecular mechanism by which apoptosis is induced in MDA-MB-231 and MCF-7 cells treated with liensinine for 24 h. In the presence of caspase inhibitor zVAD-fmk, cell viability was recovered in breast cancer cells treated with liensinine (Figure 3(a)). Western blot analysis indicated that liensinine downregulated Bcl-2 expression but did not change Bax expression in MDA-MB-231 and MCF-7 cells, indicating the decreased Bcl-2/Bax ratio. The expression levels of procaspase-3 and full length PARP were dose-dependently reduced and active caspase-3 and cleaved PARP were detected in breast cancer cells treated with 60 *μ*M liensinine (Figure 3(b)). Active caspase-3 was not detected in MCF-7 cells in this study.

### 3.4. Liensinine and Nuciferine Inhibited RANKL-Induced Osteoclast Formation and Function

**Figure 4:**
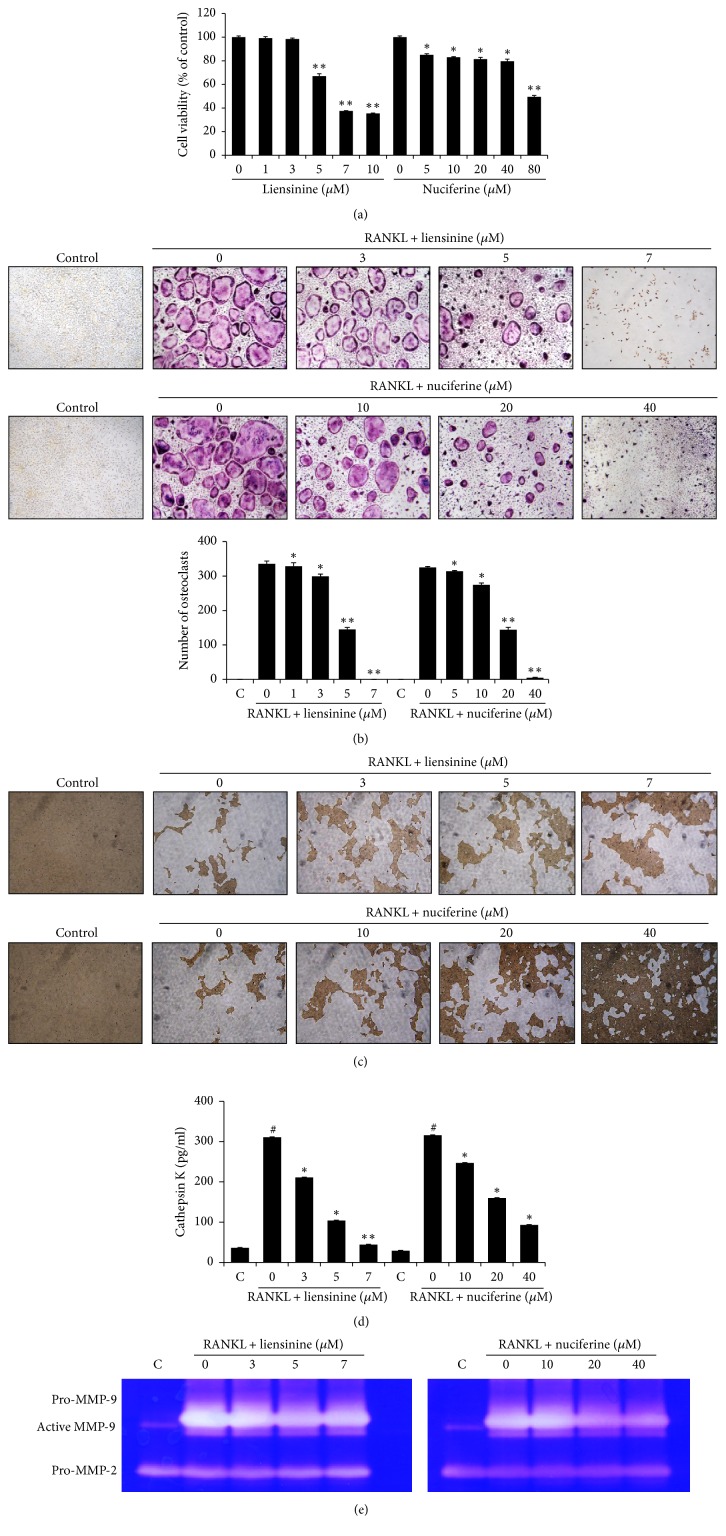
Liensinine and nuciferine inhibited RANKL-induced osteoclastogenesis and osteoclast-mediated bone resorption. (a) BMMs were incubated in 10% FBS-*α*-MEM containing M-CSF (30 ng/ml) and liensinine or nuciferine at various concentrations for 5 days. Cell viability was determined by an MTT assay. Data are expressed as the means ± SE. ^*∗*^*P* < 0.05 and ^*∗∗*^*P* < 0.001 versus BMMs without test compounds. (b) BMMs were treated with liensinine and nuciferine at the various concentrations in a presence of M-CSF and RANKL. TRAP-positive cells with >3 nuclei were counted as mature osteoclasts (100x). (c–e) BMMs were incubated in 10% FBS- *α*-MEM containing M-CSF (30 ng/ml) and RANKL (100 ng/ml) on calcium phosphate-coated plates for 5 days and then treated with liensinine or nuciferine for an additional 2 days. (c) After cell lysis, the pit area was observed under light microscopy (×100). (d) Cathepsin K levels in the collected culture media were measured using the SensiZyme Cathepsin K Activity Kit. (e) Gelatinolytic activities of MMPs in the collected culture media were detected by zymography and visualized as clear bands against the blue background. Data are expressed as the means ± SE. ^#^*P* < 0.001 versus BMMs without RANKL. ^*∗*^*P* < 0.05 and ^*∗∗*^*P* < 0.001 versus BMMs with RANKL alone.

When BMMs were incubated in media with M-CSF in the presence of different concentrations of liensinine or nuciferine for 5 days, cell viability was decreased in a dose-related manner (IC_50_ = 6.2 *μ*M for liensinine and 79.3 *μ*M for nuciferine) (Figure 4(a)). RANKL-induced osteoclast differentiation was inhibited by 57% by liensinine at 5 *μ*M and by 56% by nuciferine at 20 *μ*M. Liensinine (IC_50_ = 4.3 *μ*M) showed more potent inhibition on osteoclast formation than nuciferine (IC_50_ = 17.9 *μ*M) (Figure 4(b)).

The effects of liensinine and nuciferine on the activity of mature osteoclasts were investigated in BMMs treated with liensinine or nuciferine at noncytotoxic concentrations for 2 additional days after the induction of osteoclast differentiation by RANKL stimulation for 5 days. Treatment with liensinine or nuciferine suppressed the formation of resorption pits in a dose-related manner. In particular, nuciferine at 40 *μ*M noticeably inhibited the pit formation (Figure 4(c)). The activity of cathepsin K in the culture media was dramatically increased by RANKL stimulation but treatment with liensinine at 7 *μ*M and nuciferine at 40 *μ*M suppressed the RANKL-induced activity of cathepsin K by 86% and 71%, respectively (Figure 4(d)). The secreted levels of pro-MMP-9 and active MMP-9 were also increased in RANKL-induced mature osteoclasts but liensinine or nuciferine treatment inhibited these increases (Figure 4(e)). MMP-2 activity did not change by RANKL and two compounds.

### 3.5. Liensinine Blocked Breast Cancer-Induced Bone Loss in Mice

**Figure 5:**
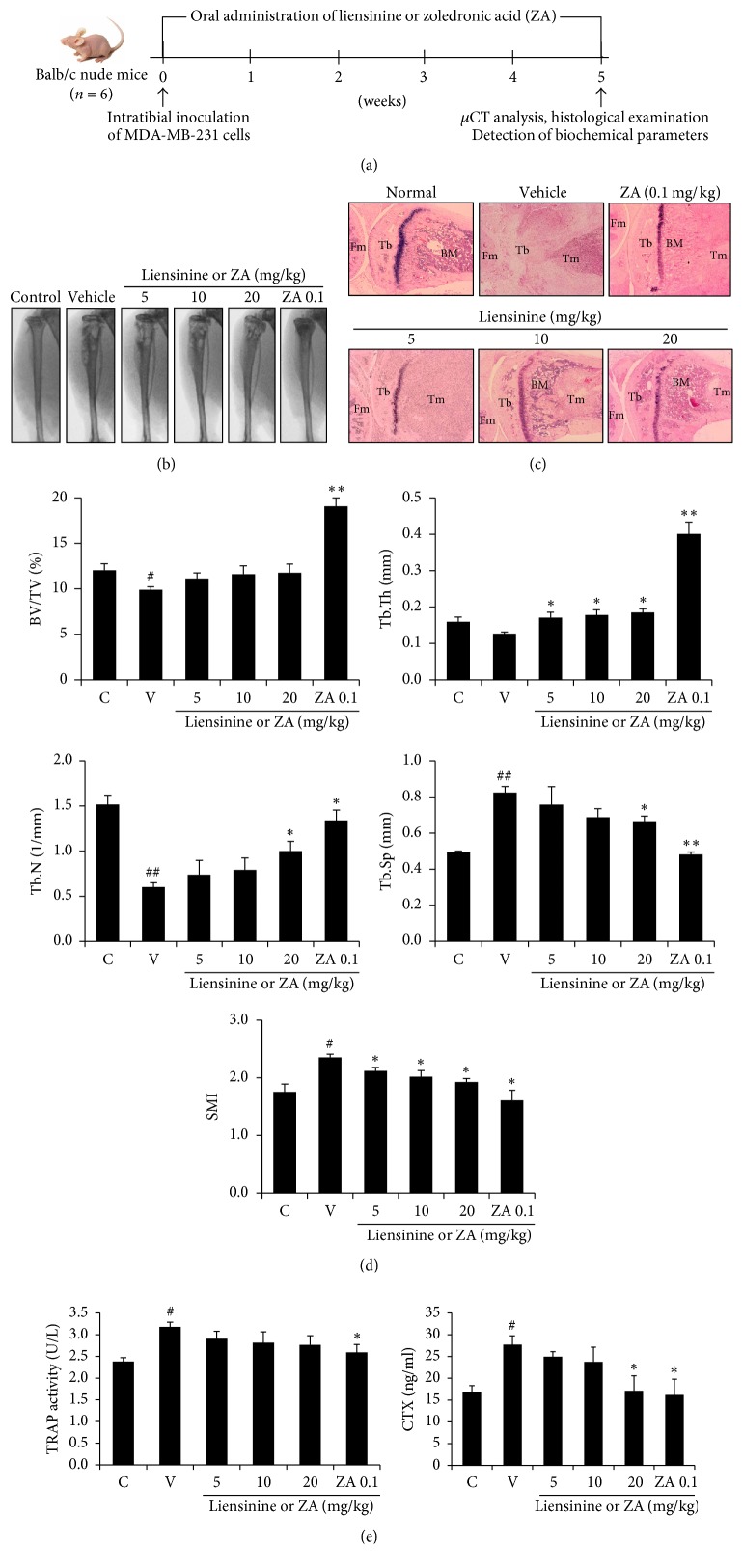
Liensinine prevented breast cancer-induced bone loss in mice. MDA-MB-231 cells were injected into the tibiae of nude mice (*n* = 6). Liensinine was administered orally five times per week and zoledronic acid (0.1 mg/kg) was injected subcutaneously 3 times per week for 5 weeks. (a) Experimental design. (b) X-ray images were taken on week 5. (c) Serial sections of mouse tibiae and femora were stained with hematoxylin-eosin. Tm: tumor mass, BM: bone marrow, Fm: femur, and Tb: tibia. (d) Bone morphometric parameters, BV/TV (%), Tb.Th (mm), Tb.N (1/mm), Tb.Sp (mm), and SMI, were analyzed with micro-CT. (e) Serum bone resorption markers, TRAP5b and CTX, were analyzed using the respective kits. Data are expressed as the means ± SE. ^#^*P* < 0.05 and ^##^*P* < 0.01 versus control mice without MDA-MB-231 cells; ^*∗*^*P* < 0.05 and ^*∗∗*^*P* < 0.001 versus cancer cell-inoculated mice with vehicle (PBS containing 1% DMSO) alone.

We investigated* in vivo* inhibitory effect of liensinine on breast cancer cell-mediated bone resorption. X-ray images indicated that severe osteolytic lesion was produced in the tibiae of mice inoculated with bone-tropic MDA-MB-231 breast cancer cells and treated with vehicle but orally administered liensinine inhibited the osteolysis (Figure 5(a)). Histological examination showed that the growth of MDA-MB-231 breast cancer cells and osteolytic progression were substantially increased in bone marrow of mice intratibially injected with MDA-MB-231 breast cancer cells. Oral administration of liensinine dose-dependently suppressed the increased tumor growth and bone destruction (Figure 5(b)). In bone morphometric analysis using micro-CT, treatment with liensinine inhibited decreases in the percentage of trabecular bone volume (BV) to total volume (TV) of bone tissue (BV/TV), the average thickness of trabecular bone (Tb.Th), and the number of intersections between bone tissue and nonbone components within a defined length of specimen (Tb.N) and increases in the average width of the trabecular bone marrow (Tb.Sp) and structure model index (SMI) as an index for the ratio of plate-like to rod-like trabecular structures, compared with vehicle treatment (Figure 5(c)). Serum levels of bone resorption markers, TRAP5b and CTX, were also inhibited in liensinine-treated mice (Figure 5(d)).

## 4. Discussion

Breast cancer has a property of metastasis to bone and bone metastasis is one of main causes of decreased survival rate in breast cancer patients. Once bone metastases grow aggressively and interact with bone microenvironment, they acquire the ability to penetrate the surrounding tissues and to resorb bone matrix, causing severe osteolysis [19]. In this study, we assessed whether liensinine and nuciferine, each marker ingredient in lotus plumules and leaves [20], could inhibit breast cancer cell-associated bone loss. The concentrations and doses for in vitro and in vivo experiments were determined based on our preliminary experimental data.

Liensinine noticeably reduced the viability, migration, and invasion of highly metastatic triple-negative MDA-MB-231 and less metastatic estrogen receptor-positive MCF-7 cells in a dose-related manner. Nuciferine significantly inhibited the viability and invasion of MDA-MB-231 cells rather than MCF-7 cells but its inhibitory activity was less potent than that of liensinine. By analyzing their inhibition levels on cell viability, migration, and invasion, we found that the reduced cell viability by liensinine and nuciferine treatment may contribute to their inhibitory activities on cell migration and invasion at noncytotoxic concentrations (less than 20 *μ*M). Because proliferation and apoptosis are the important regulatory mechanisms of cell growth [21], we further investigated whether these active compounds could induce apoptotic cell death and/or block cell proliferation. In particular, liensinine noticeably induced apoptosis in MDA-MB-231 cells rather than in MCF-7 cells. Nuciferine also induced apoptosis in both breast cancer cell lines. Necrosis was not observed in our experimental conditions. Liensinine and nuciferine inhibited BrdU incorporation into the newly synthesized DNA for cell proliferation in two breast cancer cell lines. In flow cytometric analysis, liensinine treatment clearly increased sub-G1 peak as an indicator of apoptotic cell death and induced cell cycle arrest at G2/M phase in MDA-MB-231 and MCF-7 cells compared with nuciferine treatment. These results may indicate that liensinine and nuciferine inhibit cell growth by inducing apoptotic cell death and arresting cell cycle in breast cancer cells. Liensinine may be more potent than nuciferine and more effective in the induction of apoptosis in MDA-MB-231 cells than MCF-7 cells. In addition, we confirmed that liensinine induces apoptosis via mitochondrial-dependent pathway as supported by the decreased Bcl-2/Bax ratio, the activation of caspase-3, and caspase-mediated cleavage of PARP.

When the breast cancer cells spread to the bone, cancer cell-released osteolytic factors stimulate osteoclastogenesis directly or by promoting osteoblastic/stromal RANKL expression [22]. The differentiated osteoclasts cause bone resorption by dissolving inorganic calcium hydroxyapatite in acidic conditions and organic bone matrix by MMPs and cathepsin K [23–25]. In this study, liensinine and nuciferine treatment inhibited RANKL-induced osteoclast differentiation and bone resorption by decreasing the secretion of cathepsin K and MMP-9 at nontoxic concentrations. These results suggest that liensinine and nuciferine can block RANKL-induced osteoclast formation and function. Furthermore, antibone resorptive activities of these compounds can contribute to the inhibited growth of bone metastases by blocking the release of bone-storing growth factors.

Finally, we tested the inhibitory effect of liensinine on breast cancer-mediated bone loss in the mice with intratibial injection of MDA-MB-231 cells. Oral administration of liensinine reduced osteolytic lesions in mouse tibiae, as supported by histological examination, bone morphometric parameters, and serum biochemical markers of bone resorption, TRAP5b and CTX.

Collectively, liensinine and nuciferine inhibited cell proliferation, migration, and invasion and especially induced apoptosis via mitochondrial-dependent pathway in breast cancer cells. These compounds prevented bone resorption by inhibiting the formation and function of osteoclast. Therefore, liensinine and nuciferine may be promising protective and therapeutic agents for patients with breast cancer and the related bone loss. In the further study, we will investigate whether liensinine and nuciferine can activate osteoblastic cells.
